# Alteration of m^6^A Methylation in Breast Cancer Cells by *Kalanchoe pinnata* Aqueous Extract

**DOI:** 10.3390/molecules30122634

**Published:** 2025-06-18

**Authors:** Carlos Rogelio Alvizo-Rodríguez, Fernando Calzada, Uriel López-Vázquez, Emmanuel Tomay Tiburcio, Juan A. Hernandez-Rivera, Alan Carrasco-Carballo, Marta Elena Hernández-Caballero

**Affiliations:** 1 Facultad de Medicina, Biomedicina, Benemérita Universidad Autónoma de Puebla, 13 Sur 2702 Col. Volcanes, Puebla 72410, Mexico; alvrod.cr@gmail.com (C.R.A.-R.); uriel.vazquez95@outlook.com (U.L.-V.); emmanuel.t.t.01@gmail.com (E.T.T.); 2 Unidad de Investigación Médica en Farmacología, Unidad Médica de Alta Especialidad, Hospital de Especialidades, Centro Médico Nacional Siglo XXI, Av. Cuauhtémoc 330, Colonia Doctores, Mexico City 06720, Mexico; fercalber10@gmail.com; 3 Laboratorio de Elucidación y Síntesis en Química Orgánica, Instituto de Ciencias, Benemérita Universidad Autónoma de Puebla, Puebla 72592, Mexico; juan.hernandezrivera@viep.com.mx; 4 Secretaría de Ciencia, Humanidades, Tecnología e Innovación, Laboratorio de Elucidación y Síntesis en Química Orgánica, Instituto de Ciencias, Benemérita Universidad Autónoma de Puebla, Puebla 72592, Mexico

**Keywords:** *Kalanchoe pinnata*, breast cancer, MCF-7 cells, HCC1937 cells, m^6^A, METTL3, FTO, migration

## Abstract

*Kalanchoe pinnata* is used in traditional medicine to treat cancer, as it contains flavonoids and phenols known to regulate key cellular processes associated with cancer. Breast cancer, the most common cancer among women globally, presents ongoing challenges in treatment. The discovery of m^6^A methylation and its regulation by methylosome proteins offers novel therapeutic avenues for cancer management. This study aimed to investigate the cytotoxic and epitranscriptomic effects of an aqueous extract from *K. pinnata* on MCF-7 (luminal A) and HCC1937 (triple-negative) breast cancer cells. Cell lines were treated with the aqueous *K. pinnata* extract, characterized by HPLC, for 72 h, followed by an assessment of cytotoxicity and migration. The expression of methylosome components METTL3 and FTO was measured using RT-PCR. m^6^A global methylation was assessed via colorimetry, and molecular docking studies were conducted. The results indicated that only HCC1937 cells exhibited altered migration capacity. This change was correlated in silico with the inhibition of METTL3 by luteolin and quercetin, constituents of the aqueous extract. METTL3, a methyltransferase, was overexpressed by scratch stimuli but was downregulated following *K. pinnata* treatment in both MCF-7 and HCC1937 cells. The FTO demethylase was overexpressed in both cell lines. In silico analysis suggested an interaction between FTO and compounds such as gallic acid and myricetin. Additionally, m^6^A global methylation decreased in MCF-7 cells but increased in HCC1937 cells, potentially affecting cell migration. Our findings indicate that *K. pinnata* influences both METTL3 and FTO, altering m^6^A methylation in a cell-type-dependent manner, with HCC1937 cells being particularly sensitive. Further research is required to elucidate the complete molecular mechanism of *K. pinnata*’s aqueous extract in breast cancer treatment.

## 1. Introduction

The world is home to a vast array of plants traditionally utilized for treating numerous medical conditions such as acute ulcers, wounds, burns, Leishmaniasis, fever, diabetes, and cancer [1]. Many of these plants have also contributed to the development of pharmaceuticals for specific ailments, including cancer. *Kalanchoe pinnata*, a plant used in traditional medicine, is native to Madagascar and belongs to the Crassulaceae family, which includes 1500 species distributed worldwide [2]. Fernandes et al. [3] and Elufioye et al. [4] compiled comprehensive reviews of the chemical composition of *K. pinnata*. This composition includes fatty acids, alkaloids, sterols, terpenes, flavonoids, phenols, lycopenes, tannins, vitamins, minerals, acyclic and aromatic organic acids, amino acids, sugars, bufadienolides, ketones, phenanthrenic derivatives, long-chain hydrocarbons, saponins, and gums.

Breast cancer (BC) is the most frequently diagnosed cancer among women worldwide, with nearly 2.3 million new cases and 665,675 deaths reported in 2022 according to GLOBOCAN 2022 data (Global Cancer Observatory (iarc.fr)). The incidence of BC has been increasing, particularly in transitioning countries [5]. BC can be categorized into two main types: hormone receptor-positive and hormone receptor-negative tumors. Approximately 70% of BCs exhibit high activity of the estrogen receptor (ER) and the progesterone receptor (PR). Additionally, 12–20% of cases overexpress the human epidermal growth factor receptor-2 (HER-2), while 15% are classified as triple-negative breast cancer (TNBC). TNBC is notably aggressive, exhibiting metastatic patterns and poor prognosis [6,7]. For hormone receptor-positive tumors, early screening and the use of multiple agents targeting specific receptors offer the most effective strategy for reducing mortality. In contrast, chemotherapy remains the prevalent treatment approach for hormone receptor-negative tumors.

The m^6^A modification is a dynamic and reversible chemical change that occurs at the sixth nitrogen position of adenosine (m^6^A). This modification is deposited by the RNA methyltransferase-like 3 (METTL3), which serves as the catalytic core of a seven-protein methyltransferase complex [8]. METTL3 forms a dimer with METTL14 to transfer methyl groups to specific sequences [9]. METTL3 has been shown to play a significant oncogenic role in multiple cancers, including bladder, pancreatic, hepatic, gastric, colorectal, and renal cell carcinoma (RCC). However, there are conflicting data regarding its role in RCC; some researchers consider it an oncogene, while others view it as a tumor suppressor [9,10,11].

Conversely, enzymes like the fat mass and obesity-associated protein (FTO) can remove the m^6^A modification. FTO, similar to METTL3, has been implicated as both a tumor suppressor and an oncogene. The functional variation of these enzymes is influenced by differences in downstream target genes and the type of tumor cells analyzed. For example, FTO overexpression has been shown to promote bladder cancer cell proliferation through the FTO/Mir-576/CDK6 pathway. In contrast, FTO has been identified as a suppressor in thyroid cancer [12,13,14].

The m^6^A modification has been demonstrated to influence several processes, including tumor proliferation, invasion, metastasis, stemness maintenance, and drug resistance in cancer [15,16]. Evidence suggests that combining chemotherapy with medicinal plants, such as *Astragali radix*, *Codonopsis radix*, and *Curcuma zedoaria*, can enhance treatment efficacy for patients with various cancer types [17,18] Understanding m^6^A methylation offers a novel framework for analyzing its role in complex pathologies like cancer and its interactions with drugs and natural products.

This study evaluates the cytotoxic effects of an aqueous extract of *K. pinnata* on the MCF-7 and HCC1935 BC cell lines. To achieve this, we conducted HPLC characterization and measured the phenolic and flavonoid content as well as the extract’s DPPH radical scavenging activity. Additionally, we performed a molecular docking simulation. We assessed the expression levels of the METTL3 and FTO genes and examined global m^6^A methylation following exposure to the *K. pinnata* aqueous extract, both independently and in combination with a scratch assay. This study is significant as it elucidates the role of plant extracts in modulating epigenetic factors. It also provides a foundational understanding of the potential therapeutic application of *K. pinnata* in treating diseases such as BC through the action of its secondary metabolites, including phenols and flavonoids.

## 2. Results

### 2.1. Chemical Characterization and Antioxidant Effects of K. pinnata Aqueous Extract

The aqueous extract was found to contain antioxidants. Table 1 presents the secondary metabolites identified in both the positive and negative phases.

The aqueous extract exhibited significant DPPH^•^ and ABTS^•^ radical scavenging effects, thereby reducing the activity of free radicals (Table 2). The natural antioxidants’ ability to eliminate free radicals indicates that *K. pinnata* is similarly effective in both assays, as shown by their comparable levels of inhibition. This observation suggests that the secondary metabolites responsible for neutralizing free radicals likely include phenols and flavonoids.

The total phenol content of the extract was determined to be 357.34 ± 20.64 mg EGA/kg of plant. The total flavonoid content was 408.90 ± 30.37 mg EQ/kg of plant, which corresponds to 230.16 mg EAC/kg of plant in terms of gallic acid equivalence.

### 2.2. Cytotoxic Activity of Aqueous Extract from K. pinnata on MCF-7 and HCC1937 Cell Lines

To assess the viability and cytotoxic effects of the aqueous extract from K. pinnata, we conducted an MTT assay on two cell lines. MCF-7 is a hormone-responsive BC cell line modeling estrogen receptor-positive BC [18]. HCC1937 is a BC cell line representing invasive ductal carcinoma without estrogen or progesterone receptors but with a BRCA1 mutation [19]. The cells were treated with *K. pinnata* extract at various concentrations (15, 30, 45, 60, 75, 90, and 105 µg/mL).

**Figure 1:**
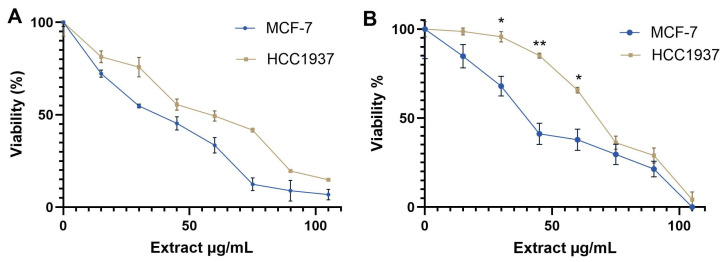
Cytotoxic effects of aqueous extract of *K. pinnata* cells after 72 h of treatment. (**A**) MTT assay, (**B**) trypan blue assay. The statistical significance was determined through the calculation of the *p*-value where the mean ± SEM of percentage cell viability of the human breast cancer cells was compared using a *t*-test. The statistical significance is represented by asterisks (* *p* < 0.05; ** *p* < 0.01).

The results showed that the extract exhibited dose-dependent cytotoxicity, reducing the proliferation of MCF-7 cells with a CC_50_ value of 40 µg/mL. In contrast, HCC1937 cells were less sensitive to the *K. pinnata* extract, with a CC_50_ value of 66 µg/mL after 72 h of incubation (Figure 1A). The trypan blue exclusion test corroborated the MTT data (Figure 1B, Tables S1 and S2).

### 2.3. Inhibition of Cell Migration on MCF-7 and HCC1937 Cells by Aqueous Extract from K. pinnata

**Figure 2:**
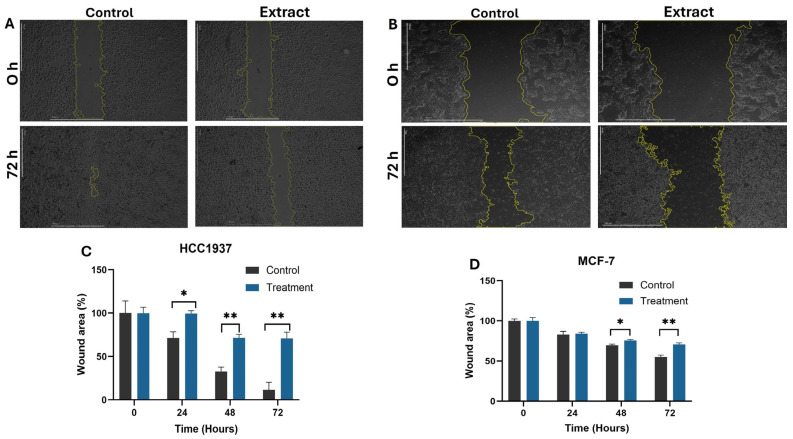
Effect of aqueous extract of *K. pinnata* on cell migration after 72 h of treatment. Microscopic images of wound healing assay in control and treated HCC1937 (**A**) and MCF-7 (**B**) cells. Photos were taken 0, 24, 48, and 72 h after scratch formation (scale bar equal to 100 µm). Percentage of wound closure in HCC1937 (**C**) and MCF-7 (**D**) cells. The statistical significance is represented by asterisks (* *p* < 0.05; ** *p* < 0.01) compared with the control without extract. The MCF-7 cells were treated with 40 µg/mL of aqueous extract, and the HCC1937 cells with 66 µg/mL.

Cancer progression is marked by cell migration, often modeled through incision wounds. In our study, wound healing significantly affected the migration behavior of HCC1937 cells compared to MCF-7 cells. The wound healing percentage in the HCC1937 treatment group showed a notable reduction compared to MCF-7, indicating that the aqueous extract decreases the mobility of metastatic HCC1937 cells (Figure 2A–C). This reduction did not occur in MCF-7 cells, which displayed more unhealed areas at the end of the treatment period (Figure 2B–D).

### 2.4. Effect on METTL3 Expression in HCC1937 and MCF-7 Cells by Aqueous Extract from K. pinnata and Mechanical Stimulus

**Figure 3:**
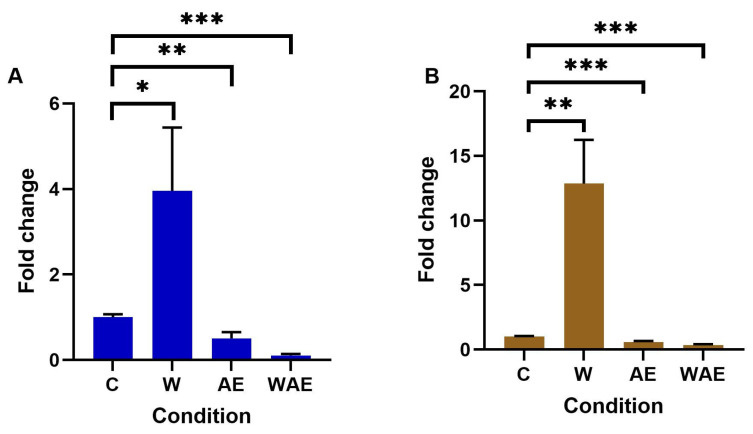
Changes in METTL3 expression after treatment with aqueous extract of *K. pinnata* in (**A**) MCF-7 and (**B**) HCC1937 cells. A higher expression was observed in HCC1937 cells after the wound was performed but with no effect from the aqueous extract only. The non-treated group was the control (C), along with a wound healing group (W), an aqueous extract group (AE), and a wound healing and aqueous extract group (WAE) (* *p* < 0.05; ** *p* < 0.01, *** *p* < 0.001). The MCF-7 cells were treated with 40 µg/mL of aqueous extract, and the HCC1937 cells with 66 µg/mL.

METTL3 is the protein responsible for adding m^6^A to mRNAs, thereby regulating gene expression. This study examined METTL3 expression in cell lines using quantitative reverse transcription polymerase chain reaction (RT-qPCR). The results indicated that METTL3 expression levels increased following wounding in both cell lines, with the highest levels in HCC1937 cells (Figure 3B). The aqueous extract influenced METTL3 expression, as transcript levels significantly decreased in both cell lines when they were scratched and treated with the extract (Figure 3B).

### 2.5. Effect on FTO Expression in HCC1937 and MCF-7 Cells by Aqueous Extract from K. pinnata and Mechanical Stimulus

**Figure 4:**
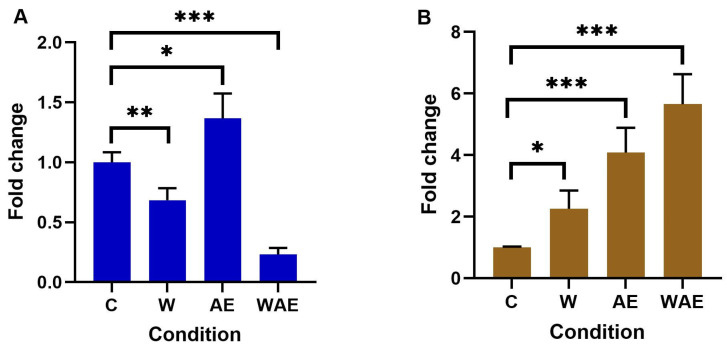
Changes in FTO expression after treatment with aqueous extract of *K. pinnata* in (**A**) MCF-7 and (**B**) HCC1937 cells. The non-treated group was the control (C), along with a wound healing group (W), an aqueous extract group (AE), and a wound healing and aqueous extract group (WAE), (* *p* < 0.05; ** *p* < 0.01, *** *p* < 0.001). The MCF-7 cells were treated with 40 µg/mL of aqueous extract, and the HCC1937 cells with 66 µg/mL.

One of the two proteins responsible for removing the m^6^A mark and participating in processes such as proliferation, migration, and invasion was analyzed. Our research revealed that FTO was overexpressed in both cell lines. In MCF-7 cells, significant changes were observed, although these changes did not reach a twofold increase (Figure 4A). Higher FTO expression was noted in HCC1937 cells (Figure 4B). Additionally, FTO expression increased in HCC1937 cells treated with the aqueous extract of *K. pinnata* and the wound.

### 2.6. Effect on m^6^A Levels in MCF-7 and HCC1937 Cells by K. pinnata Extract

**Figure 5:**
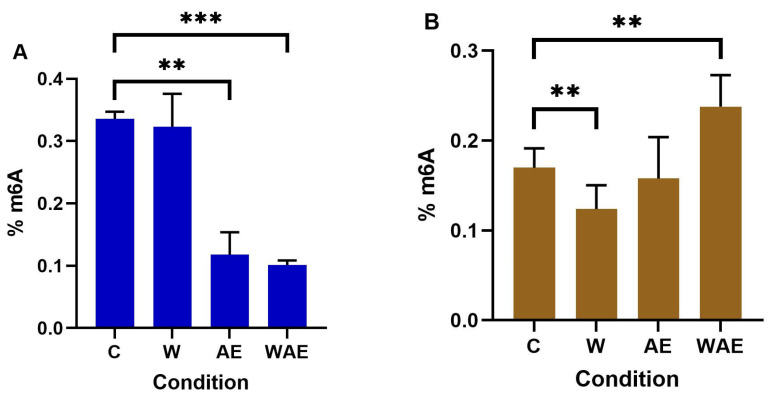
Effect of aqueous extract of *K. pinnata* on m^6^A global methylation. The m^6^A levels in (**A**) MCF-7 and (**B**) HCC1937 cells were detected using a m^6^A RNA kit. The data represent the average of three independent experiments (mean ± SEM). The non-treated group was the control (C), along with a wound healing group (W), an aqueous extract group (AE), and a wound healing and aqueous extract group (WAE), (* *p* < 0.05; ** *p* < 0.01, *** *p* < 0.001). The MCF-7 cells were treated with 40 µg/mL of aqueous extract, and the HCC1937 cells with 66 µg/mL.

In a comparative analysis of global m^6^A levels in MCF-7 and HCC1937 cell lines, it was observed that MCF-7 control cells initially exhibited higher levels of the m^6^A mark, which subsequently decreased following treatment. Notably, significant differences were found between the control group and the three intervention groups within the MCF-7 cells. The application of the aqueous extract of *K. pinnata* led to a significant reduction in m^6^A levels, an effect that was more pronounced in the presence of the scratch stimulus (Figure 5A). Conversely, in HCC1937 cells, exposure to the aqueous extract of *K. pinnata* did not result in a statistically significant change. However, the introduction of the scratch stimulus alone led to a significant decrease in m^6^A levels, while its presence alongside the extract increased the percentage of m^6^A (Figure 5B).

### 2.7. Molecular Docking Analysis

The 14 bioactive compounds derived from *K. pinnata* were characterized and analyzed as targets for METTL3 and FTO proteins using molecular docking (Table 3). Our analysis revealed that none of the compounds within the extract surpassed the docking scores of the reference inhibitors. Among the compounds docked against METTL3, luteolin and quercetin, both flavonoids, exhibited the highest energies. Their aromatic structures and hydroxyl groups mimic the hydrogen bonds found in the naturally occurring inhibitor adenosine, accounting for their elevated docking scores. However, a greater number of polar groups does not necessarily correlate with higher docking energies. For instance, the presence of an additional hydroxyl group at position 5′ in myricetin leads to a significant reduction in docking score, from −6.970 in quercetin to −3.933 for myricetin. In the context of FTO, the best docking scores were achieved by gallic acid (−8.210 kcal/mol) and myricetin (−8.163 kcal/mol). Gallic acid’s small size and three hydroxyl groups facilitate direct interaction with catalytic residues, whereas larger structures impede their interaction with binding sites, resulting in lower docking scores compared to the experimental inhibitory molecule.

## 3. Discussion

Despite considerable efforts, therapeutic strategies for treating hormone receptor-positive and hormone receptor-negative tumors—including hormonal therapy, chemotherapy, surgery, and radiation therapy—remain limited, particularly for hormone receptor-negative tumors. Systemic treatments often lack selectivity, leading to toxicity in healthy cells and resistance to anticancer agents. Although more precisely targeted drug therapies have improved efficacy, they have also increased financial costs. Therefore, combining these therapies with natural products may enhance the effectiveness of chemotherapeutic agents while potentially reducing costs.

Using two BC cell lines, MCF-7 and HCC1937, we examined the effect of the aqueous extract of *Kalanchoe pinnata* leaves in vitro. Our study focused on analyzing the cytotoxic, proliferative, and epitranscriptomic actions of *K. pinnata* (known as *Bryophyllum pinnatum*). The extract exhibited a significant, dose-dependent cytotoxic effect on MCF-7 cells compared to HCC1937 cells and demonstrated growth inhibition, particularly in MCF-7 cells, which might be attributed to the differences between these cell lines.

Ramon et al. [20] identified flavonoids such as quercetin, kaempferol, apigenin, and epigallocatechin gallate in the aqueous *K. pinnata* extract. Flavonoids are known for their beneficial effects in various diseases, including cardiovascular and neurodegenerative disorders, as well as cancer. These compounds exhibit strong pro-oxidant activity, promoting apoptosis in cancer cells, cell cycle inhibition, autophagy, regulation of inflammatory signaling, and reduction of invasiveness [21]. Other compounds in *K. pinnata*, such as phenols (e.g., gallic acid, caffeic acid, *p*-coumaric acid), also contribute to cell cycle arrest, apoptosis, and reduced invasiveness [22,23,24].

Our analysis of the *K. pinnata* aqueous extract had a phenol content of 357.34 ± 20.64 mg EGA/kg of plant and total flavonoids of 408.90 ± 30.37 mg EQ/kg of plant. This was confirmed by the extract analysis by HPLC-QtoF-MS. These findings align with the existing literature, highlighting phenolic compounds with antioxidant properties. Thus, the aqueous extract’s composition supports its cytotoxic activity consistent with results obtained by other researchers in various tumoral cell lines, including Caco-2, T84, A2058, HeLa, HL-60, and U87-MG cells [25,26].

Relatively few studies have used the aqueous extract of *K. pinnata*, with some including related species such as *K. blossfeldiana* or *K. daigremontiana*, noted for their potent cytotoxic, antioxidant, or anti-inflammatory activities [27,28,29]. The effect of the *Kalanchoe* genus on cell migration is underexplored, though *K. tubiflora* has demonstrated moderate wound healing effects with cellular cytotoxicity in mouse fibroblast 3T3 migration and proliferation assays, as reported by Schmidt et al. [30]. Hsieh et al. [31] found that an n-Butanol-soluble fraction from lung carcinoma cells A-549 can inhibit cell migration.

In our study, the aqueous extract of *K. pinnata* significantly affected the migration of HCC1937 cells more than MCF-7 cells, although MCF-7 cells’ survival was more compromised by cytotoxicity, with noticeable changes in migration occurring up to 48 h. HCC1937 cells possess a BRCA1 mutation; Liao et al. [32] found that this mutation induces translocation of β-catenin to the nucleus and deregulation of Wnt/β-catenin. Other studies indicate that compounds like quercetin, kaempferol, epigallocatechin-3-gallate, and apigenin reduce cell migration and invasion in carcinoma cell lines by regulating genes involved in pathways such as ubiquitination, PI3K/Akt, MAPK, JAK/STAT, NF-κB, Wnt/β-catenin, or Notch [33,34,35]. Recently, Li et al. highlighted that METTL3 upregulation can regulate contractile gene expression [36].

Epigenetic regulation is a critical target in cancer treatment because, unlike genetic mutations, epigenetic modifications are reversible. Numerous studies have focused on DNA methylation and histone modifications, revealing that many genes are hypomethylated and overexpressed, whereas others are hypermethylated and underexpressed. A few decades ago, researchers discovered that RNA undergoes epigenetic modification, with the m^6^A modification being one of the most studied. This modification involves various mRNA modulations, and its addition is mediated by a group of proteins, including the methyltransferase METTL3.

Few studies have explored the behavior of methylosome components in response to treatments with polyphenolic and flavonoid-rich extracts. Our study evaluated the effect of an aqueous extract of *Kalanchoe pinnata* on METTL3 expression, both with and without wound healing assay stimuli. We observed that the *K. pinnata* extract reduced METTL3 transcript expression in MCF-7 and HCC1937 cells. In contrast, the wound healing stimulus increased METTL3 transcript levels in both cell lines, more notably in HCC1937 cells, with a fold change of 13. However, when both stimuli were combined, METTL3 transcript levels decreased significantly, almost to zero. This finding supports the role of METTL3 in tumor cell proliferation and migration and demonstrates how the *K. pinnata* extract can modulate METTL3 expression, reducing migration in HCC1937 cells compared to MCF-7 cells. We propose that the greater gene alteration in the metastatic HCC1937 cell line explains the extract’s pronounced effect, given its more aggressive phenotype.

Research indicates that METTL3 facilitates tumor progression by regulating genes such as Bcl-2, EPPK1, and ACIN1 [37,38,39,40]. Studies in chicken liver cancer, mouse myoblasts, pancreatic carcinoma, and human liver cells have noted that quercetin acts as a METTL3 inhibitor, decreasing m^6^A levels [41,42,43]. For instance, Li et al. [36] found that while quercetin did not influence METTL3 protein levels, it significantly reduced m^6^A levels in vascular smooth muscle cells. These findings underscore the significance of dose dependency and the specific cell type. Our molecular docking analysis suggests that quercetin and luteolin could have inhibitory effects on METTL3. To date, there is no evidence that luteolin decreases METTL3 levels, although it has demonstrated antitumor effects on A375 melanoma cells [44].

We investigated the alterations in FTO, a demethylase responsible for removing the m^6^A modification from mRNAs. Our findings indicate that FTO overexpression does not enhance cellular migration in HCC1937 cells, unlike in Panc-1, SW1990, or gastric adenocarcinoma cell lines, as previously reported [45,46]. Some researchers have observed that FTO overexpression may reduce migration in epithelial cancers [47]. While the expression of METTL3 was significantly reduced by the aqueous extract, FTO expression increased in HCC1937 cells treated with the extract and was further elevated with both the extract and scratch stimuli. Despite this increased expression, cell migration slowed. Molecular docking analysis revealed that gallic acid and myricetin might regulate the FTO protein. In contrast, changes in FTO and METTL3 gene expression in MCF-7 cells did not result in notable effects on cell migration, which were only observed in HCC1937 cells. Considering that FTO is an RNA demethylase frequently upregulated in many tumors and represents a risk factor for overall survival, *K. pinnata* emerges as a potential therapeutic option for aggressive cancers, such as hormone receptor-negative tumors.

To date, few studies have examined the impact of plant extracts on m^6^A levels. m^6^A methylation predominantly occurs in the mRNA coding sequence, 3′-UTR, and stop codon, influencing mRNA transcription, splicing, nuclear export, translation, and degradation [48]. METTL3 and FTO play crucial roles in the addition and removal of the m^6^A methylation mark. This study aimed to evaluate the effect of *K. pinnata* on global m^6^A levels. After treating MCF-7 cells with *K. pinnata* aqueous extract, we observed a correlation between METTL3 and FTO expression and a decrease in global m^6^A levels (from 0.33% to 0.11%). However, with the introduction of a scratch stimulus, m^6^A levels remained low, but the correlation with FTO was lost due to a significant reduction in FTO expression.

In HCC1937 cells subjected to a simulated wound, m^6^A levels decreased (from 0.17% to 0.12%), which was corroborated by an increase in FTO. Upon adding the extract, m^6^A levels increased to 0.15%, although this was not reflected in significant changes to m^6^A levels compared to the control. With both stimuli, FTO and m^6^A levels increased further to 0.23%. In this context, m^6^A levels were inconsistent with FTO or METTL3 expression. Schwanhäusser et al. [49] suggest that reduced m^6^A methylation may prolong the lifespan of new RNAs, as cellular protein abundance is regulated at the translational level. Our findings indicate that increased m^6^A could be influencing how reader proteins regulate the translation of proteins involved in migration, a conclusion supported by other studies.

## 4. Materials and Methods

### 4.1. Preparation of Plant Extract

#### 4.1.1. Collection

Fresh leaves of *K. pinnata* (Lam.) Pers were collected by some of the authors (Alvizo, Hernández, and López) in Cosautlán de Carvajal, Veracruz, México. M. en C. Santiago Xolalpa, an expert from the Herbarium of CMN SXXI, IMSS, identified the plant, assigning it voucher No. 16963. The specimen was deposited in the Herbarium IMSSM at the Instituto Mexicano del Seguro Social.

#### 4.1.2. Dry and Extraction

The leaves were cleaned to remove any foreign matter and then dried for 2 weeks in a desiccator set at 35 °C. Subsequently, they were ground using a mortar to obtain a fine powder. This powder was stored in a glass bottle and kept in a dry place until the extraction process began. Extraction was performed by Soxhlet extraction for 3 h with water as the solvent. The extract was then removed from the equipment and diethyl ether was added to promote water distillation. The same was carried out by high vacuum evaporation at 45 °C to minimize metabolite decomposition at high temperatures. The resulting dry extract was stored at 4 °C until use.

#### 4.1.3. HPLC-QToF-MS Analysis

The sample was prepared as follows: 100 mg of the dry extract, described above, was placed with 1 mL of methanol, and sonicated for 30 min. It was centrifuged for 15 min at 11,000 rpm and passed through a 0.22 µm PFTE filter. The analysis was carried out on an Agilent Infinitum 1260II HPLC (Agilent. Santa Clara, CA, USA). The separation of the sample components was carried out in a Phenomenex Luna 5 µm C18 column (Phenomenex, Torrance, CA, USA), 4.6 × 250 mm, at 40 °C and with 0.7 mL min^−1^ of mobile phase consisting of A: Water and B: Acetonitrile, both with 0.1% formic acid. The separation gradient started with 5% B and reached 15% B in 40 min; at 65 min, it reached 60% B; at 75 min, it reached 95% B and remained there until 80 min. The gradient was then returned to the initial % B and maintained for 10 min. ESI-QTOF-MS 6520 detector (Agilent) was used in positive and negative ionization modes with the following source parameters: fragmentor voltage 175 V, capillary voltage 3500 V, gas temperature 350 °C, N2 low rate 11 L min^−1^, and nebulizer pressure of 60 psi.

### 4.2. Antioxidant Tests

#### 4.2.1. DPPH Assay

The DPPH assay was adapted for use with a 96-well microplate with some modifications. To each well of the ELISA plate, 25 µL of the sample and 200 µL of a DPPH solution (initial absorbance of 0.750) were added. The plate was then incubated in the dark for 40 min. Absorbance was measured using an ELISA microplate reader set at a 510 nm filter. The percentage of inhibition was determined by:

$$\%\;inhibition=\frac{Abs\;ref-Abs\;extract}{Abs\;ref}\times 100$$

#### 4.2.2. ABTS Assay

In the ABTS assay, the reaction initially involved a 2.45 mM potassium persulfate solution to generate the free radical through a 16-h process. Following this, 20 µL of the sample and 280 µL of an ABTS solution (with an initial absorbance of 0.700) were added to each well of the ELISA plate. The mixture was incubated in the dark for 40 min. Afterward, absorbance was measured using an ELISA microplate reader equipped with a 760 nm filter. The inhibition percentage was calculated by the equation above.

#### 4.2.3. Folin–Ciocalteu Assay

The determination was conducted using the Folin–Ciocalteu reagent methodology. We added 150 µL of the sample, 2250 µL of distilled water, 150 µL of the Folin–Ciocalteu reagent, and 450 µL of 12% Na_2_CO_3_ to each cell. In this part of the study, we included a positive control (gallic acid) and a blank. The mixture was incubated in darkness for 2 h. Subsequently, we measured the absorbance at 760 nm using a UV-Vis spectrophotometer.

### 4.3. Flavonoid Total Test

Four milliliters of distilled water were added to a test tube, followed by 1 mL of the sample. Each subsequent addition was homogenized using a vortex mixer. Next, 0.3 mL of NaNO_2_ (1 M), 0.3 mL of AlCl_3_ (10%), and 2 mL of NaOH (1 M) were added. The mixture was then topped up to a total of 10 mL with distilled water. The tube was incubated in the dark for 40 min. Subsequently, 3 mL of the contents was transferred to a cuvette, and the absorbance was measured spectrophotometrically at 510 nm.

### 4.4. Cell Culture

The human BC MCF-7 cell line, which serves as a model for estrogen receptor-positive BC [50], and the HCC1937 cell line, representing invasive ductal carcinoma without estrogen and progesterone receptors but with a BRCA1 mutation [19], were cultured in Dulbecco’s Modified Eagle’s Medium (DMEM, Gibco, Thermo Fisher, Waltham, MA, USA) and RPMI (Gibco, Thermo Fisher, USA), respectively. The media were supplemented with 100 U/mL of penicillin, 100 mg/mL of streptomycin, 0.25 µg/mL (Gibco, Thermo Fisher, USA), and 10% (*v*/*v*) fetal bovine serum (FBS, Biowest, Nuaillé, France). The cells were incubated at 37 °C in an atmosphere of 5% CO_2_.

### 4.5. Staining with Trypan Blue

To analyze cellular viability using a trypan blue exclusion assay, we treated MCF-7 and HCC1937 cell lines with *K. pinnata*. Approximately 200,000 cells from each cell line were seeded into 24-well plates. They were then exposed to aqueous extract concentrations ranging from 0 to 105 µg/mL for 72 h. After washing with PBS 1X, the cells were trypsinized. A 0.4% trypan blue stain (Omnichem, Wetteren, Belgium) was added to MCF-7 with DMEM and to HCC1937 with RPMI, using a 1:1 dilution of trypan blue. Cell viability was assessed by counting cells in a hemocytometer. The cells were again diluted (1:1), applied to the hemocytometer, and counted using a 10× objective. The experiments were conducted in triplicate, with results expressed as mean ± SEM.

### 4.6. MTT Assay

To estimate the cytotoxic effect of the aqueous fraction, we conducted an MTT (3-[4,5-dimethylthiazol-2-yl]-2,5-diphenyltetrazolium bromide; Sigma Aldrich, St. Louis, MO, USA) assay on two cancer cell lines: MCF-7 and HCC1937. The cell lines were seeded in 96-well plates at a density of 1 × 10^4^ cells per well. One day post-seeding, the cells were exposed to the aqueous plant fraction at concentrations ranging from 0 to 105 µg/mL. After 72 h of exposure, MTT at a concentration of 0.5 mg/mL was added to the cells and incubated for 4 h. The absorbance of the resulting formazan solution, dissolved in isopropanol with 0.01N HCl, was measured using a microplate reader at 570 nm (Accuris, BioTek Benchmark Scientific, Sayreville NJ, USA). Data were analyzed using linear regression and expressed as cytotoxic concentration (CC_50_) mean values (±SEM) from two independent experiments, each performed in eight repetitions (*n* = 48).

### 4.7. Wound Healing Assay

The cells were seeded in 6-well plates and allowed to grow until they reached 80% confluence. We included both a group treated with the aqueous extract of *K. pinnata* and a non-treated control group. Once the cells achieved confluence, a straight scratch was made on the BC cell monolayer using a 200 µL sterile pipette tip, with a grid design to ensure consistent field acquisition for imaging. Subsequently, the cells were washed with PBS 1X to remove cell debris. Photographs of the scratch wound were taken every 24 h, from 0 to 72 h, to monitor cell migration into the denuded area. Digital images were captured using an inverted microscope with a 4× objective (Motic AE2000, Ca, Motic Instruments, Inc., Richmond, BC, Canada). The scratch area was analyzed using Image-J software to calculate the percentage of area reduction (wound closure). All experiments were conducted in triplicate, and results were expressed as mean ± SEM.

### 4.8. RNA Extraction and Quantitative Real-Time PCR

The cells were seeded in 6-well plates and cultured until they reached 80% confluence. We included the following groups: a non-treated control group (C), a wound healing group (W), an aqueous extract group (AE), and a combined wound healing and aqueous extract group (WAE). The wound healing assay was conducted as described previously. Following treatment, total RNA was isolated from the cells using Trizol reagent (Invitrogen, Carlsbad, CA, USA) following the manufacturer’s protocol. The quality and quantity of the extracted RNA were assessed using a Nanodrop spectrophotometer (Thermo Fisher Scientific, Waltham, MA, USA). cDNA was synthesized from this RNA using the ImProm-II Reverse Transcription System (Promega, San Luis Obispo, CA, USA). Quantification of RNA levels was executed on a Step One real-time PCR system (Kapa Biosystems Pty, Wilmington, MA, USA). The cDNA served as a template for gene expression analysis using the Kapa SYBR Fast qPCR kit (Applied Biosystems, San Francisco, CA, USA). Primer sequences were as follows: HPRT forward primer ACACTGGCAAAACAATGCAGA, reverse primer ATATCCAACACTTCGTGGGGT (110 bp); FTO forward primer TGGCTCAACTGGAAGCACTG, reverse primer CAGTGAGCGAGGCAAGGATG (132 bp); METTL3 forward primer GCTCTATCCAGGCCCACAAG, reverse primer TGGAGACAATGCTGCCTCTG (116 bp). HPRT was used as an internal control. Each reaction was conducted in triplicate, and data were analyzed by comparing Ct values. All PCR primers were procured from Integrated DNA Technologies (IDT, Coralville, IA, USA).

### 4.9. m^6^A Assay

Total RNA was isolated from cells according to a previously described method. The m^6^A RNA Methylation Assay Kit Colorimetric (Abcam, Cambridge, UK) was used to determine the absolute N6-methyladenosine modification levels, following the manufacturer’s protocol. Briefly, 200 ng of RNA was added to strip wells for binding, then washed and incubated with a capture antibody. The m^6^A was subsequently detected, and absorbance was read at 450 nm. Each sample was analyzed in triplicate.

### 4.10. Molecular Docking Study

The bioactive compounds obtained from the HPLC-QToF-MS characterization of *K. pinnata* water extract were prepared and optimized using Macromodel (https://www.schrodinger.com/citations/, accessed on 27 May 2025), employing water as a solvent, with an RMSD allowance of less than 0.3 Å. Subsequently, physiological conditions were simulated at pH 7.4 through tautomer analysis in LigPrep (https://www.schrodinger.com/citations/, accessed on 27 May 2025), using OPLS4 as the force field. The proteins FTO and MTTL3 were prepared within the protein preparation wizard module (https://www.schrodinger.com/citations/, accessed on 27 May 2025) at pH 7.4. This preparation involved optimizing hydrogen bonds and minimizing structures to 0.3 Å, followed by generating the grid box at the catalytic sites of both proteins. Molecular docking simulations were conducted using the Glide module (https://www.schrodinger.com/citations/, accessed on 27 May 2025) in the Schrodinger computational platform, adhering to the previously established protocol [51] with an OPLS4 force field. The results were analyzed at the docking score level for comparison with reference inhibitors.

### 4.11. Statistical Analysis

Data are presented as mean ± SEM. All statistical analyses were conducted using GraphPad Prism version 10.2.3.403 (GraphPad Software, Inc., San Diego, CA, USA). Results were deemed significant at a *p*-value of less than 0.05.

## 5. Conclusions

The aqueous extract of *K. pinnata* can decrease the migration capacity of HCC1937 cells by reducing the expression of METTL3, with key components like luteolin and quercetin contributing to this effect. This finding is significant given that cancer remains a leading cause of death and conventional therapies often suffer from high toxicity and a lack of selectivity. Therefore, *K. pinnata* presents a potential alternative as an adjuvant treatment. While the expression of the m^6^A regulator FTO did not fully correlate with m^6^A levels, our evidence indicates that *K. pinnata* can influence global RNA methylation and potentially impact the migration of cells with metastatic characteristics, such as HCC1937. The high levels of flavonoids and phenols in the extract support its use, highlighting the advantages of utilizing multiple active compounds—as practiced in traditional medicine—rather than isolated molecules.

## Figures and Tables

**Table 1:** Chemical structure of secondary metabolites detected by HPLC-QToF-MS.

Name	Formula	[M + H]^+^	Polarity
Apigenin	C_15_H_10_O_5_	271.0601	Positive
Luteolin	C_15_H_10_O_6_	287.0550	Positive
Diosmetin	C_16_H_12_O_6_	301.0707	Positive
Quercetin–Methylester	C_16_H_12_O_7_	317.0656	Positive
Astragalin	C_21_H_20_O_11_	449.1078	Positive
Rutin	C_27_H_30_O_16_	611.1607	Positive
		**[M − H]** *^−^* **^.^**	
*p*-coumaric acid	C_9_H_8_O_3_	163.0400	Negative
Gallic acid	C_7_H_6_O_5_	169.0142	Negative
Caffeic acid	C_9_H_8_O_4_	179.0350	Negative
Syringic acid	C_9_H_10_O_5_	197.0455	Negative
Kaempferol	C_15_H_10_O_6_	285.0405	Negative
Quercetin	C_15_H_10_O_7_	301.0354	Negative
Myricetin	C_15_H_10_O_8_	317.0303	Negative
Bryophyllin B	C_26_H_34_O_9_	489.2130	Negative

**Table 2:** Scavenging DPPH and ABTS for *K. pinnata* aqueous extract.

Concentration mg/mL	Inhibition % DPPH	Inhibition % ABTS
10	77.275 ± 1.185	88.979 ± 0.533
8	75.170 ± 0.564	85.154 ± 0.705
6	64.699 ± 1.627	73.905 ± 1.924
4	48.995 ± 0.476	59.856 ± 2.394
2	34.073 ± 0.743	43.659 ± 2.128

**Table 3:** Docking scores (kcal/mol) of secondary metabolites present in *K. pinnata* against METTL3 and FTO proteins.

Molecule	METTL3	FTO
Apigenin	−6.649	−6.62
Luteolin	−6.792	−6.562
Diosmetin	−6.872	−7.324
Astragalin	−5.262	−5.69
Rutin	−6.521	−5.406
*p*-coumaric acid	−5.701	−7.395
Gallic Acid	−4.076	−8.21
Caffeic Acid	−6.119	−7.146
Syringic Acid	−6.131	−7.444
Kaempferol	−4.008	−6.919
Quercetin	−6.970	−7.500
Myricetin	−3.933	−8.163
Bryophyllin B	−4.601	−3.212
Quercetin methyl	−6.43	−7.601
Adenosine	−7.553	
6MK		−8.259

## Data Availability

The original contributions presented in this study are included in the article/Supplementary Material. Further inquiries can be directed to the corresponding authors.

## Supplementary Materials

The following supporting information can be downloaded at: https://www.mdpi.com/article/10.3390/molecules30122634/s1, Table S1: MTT assay; Table S2: Trypan blue assay.

## Abbreviations

The following abbreviations are used in this manuscript:

BC	Breast cancer
ER	Estrogen receptor
PR	Progesterone receptor
HER-2	Human epidermal growth factor receptor-2
TNBC	Triple-negative breast cancer
METTL3	Methyltransferase-like 3
FTO	Fat and obesity-related protein
RCC	Renal cell carcinoma
SEM	Standard error of media
NT	Nontreated group
W	Wound healing group
AE	Aqueous extract group
WAE	Wound healing and aqueous extract group
