# Supplementary material for: Alteration of m6A Methylation in Breast Cancer Cells by Kalanchoe pinnata Aqueous Extract
Source: Molecules. 2025 Jun 18;30(12):2634. doi: 10.3390/molecules30122634 (PMC12195708; doi:10.3390/molecules30122634)
Supplement: Supplementary file 1 [file molecules-30-02634-s001.zip › molecules-3696737-supplementary.pdf]

Table S1. MTT assay

HCC1937 cells		
Extract μg/mL	Viability (%)	SE
0	100	4.01
15	87.11	1.92
30	78.25	2.71
45	77.02	3.08
60	67.25	2.26
75	57.20	2.80
90	28.22	14.17
105	22.53	3.71
MCF-7		
0	100	7.43
15	72.33	3.23
30	54.79	5.22
45	45.40	3.02
60	33.58	2.79
75	12.49	1.00
90	8.98	0.14
105	6.89	0.56

Table S2. Trypan blue assay

HCC1937 cells		
Extract μg/mL	Viability (%)	SE
0	100	4.35
15	98.67	1.91
30	95.64	2.90
45	85.10	1.40
60	65.69	1.62
75	36.17	3.72
90	28.98	4.18
105	4.255	4.26
MCF-7		
0	100	16.61
15	84.77	6.62
30	67.90	5.57
45	41.15	5.94
60	37.86	5.94
75	29.63	5.70
90	21.40	4.36
105	0	-
